# Improvement Following Housing Stabilization in a Person Experiencing Homelessness With Chronic Venous Insufficiency and Stasis Dermatitis

**DOI:** 10.7759/cureus.108497

**Published:** 2026-05-08

**Authors:** Stephanie Sutton, Pia Valvassori

**Affiliations:** 1 Internal Medicine, University of Central Florida College of Medicine, Orlando, USA; 2 Medical Education, University of Central Florida College of Medicine, Orlando, USA; 3 Family Medicine, Health Care Center for the Homeless, Orlando, USA

**Keywords:** dermatitis, housing, people experiencing homelessness, social determinants of health, venous insufficiency, wound healing

## Abstract

Chronic venous insufficiency with stasis dermatitis is a common condition that requires careful dermatologic management, including wound care and compression therapy. Management can be particularly challenging for people experiencing homelessness. This report details the course of a man previously experiencing homelessness whose stasis dermatitis and overall health improved following placement in a permanent supportive housing program. Addressing social determinants of health, including housing, hygiene, and care access, facilitated healing, highlighting housing stabilization as an essential component of effective dermatologic management.

## Introduction

Chronic venous insufficiency with stasis dermatitis is a common dermatologic condition driven by venous hypertension. Its clinical course may progress from edema and eczematous skin changes to lichenification, lipodermatosclerosis, and venous ulceration [1]. Management is multifactorial, including exercise, leg elevation, compression therapy, and wound care. Nonetheless, long-term outcomes following ulceration remain poor, with a five-year recurrence rate of approximately 25% to 50%, depending on initial severity. Moreso, ulcer closure does not necessarily indicate return to function, pain resolution, or improved quality of life. If the underlying venous insufficiency is not addressed, patients remain at risk for progressive disease, persistent erythema, and chronic nonhealing ulcerations.

These challenges are exacerbated among people experiencing homelessness (PEH), who face barriers to dermatologic and wound care, including limited access to hygiene facilities, transportation, and medical follow-up [2]. Consequently, PEH experience delayed healing and higher rates of recurrent infections [3]. Population-level data indicate that homelessness is associated with an increased incidence of diagnosed dermatologic conditions and greater use of emergency and non-dermatologic care settings [4]. Housing status is a fundamental social determinant of health (SDOH), and interventions that reduce or eliminate homelessness can improve chronic disease management, quality of life, and health care utilization [3,5].

This report details the dermatologic course of a man with chronic venous insufficiency complicated by stasis dermatitis and ulcerations whose wound healing and overall health improved following placement in a shelter, first an emergency shelter, later a housing stabilization program, in Orlando, Florida. This case illustrates the impact of housing stability on health outcomes, demonstrating that addressing social determinants, especially housing, can enhance recovery, reduce complications, and reduce health care utilization.

## Case presentation

A 53-year-old Black male experiencing homelessness presented with chronic venous insufficiency, complicated by a multi-year history of edema, stasis dermatitis, hyperpigmentation, recurrent cellulitis, and non-healing ulcers. He was evaluated at a street medicine clinic co-located within a drop-in center serving PEH. His past medical history is notable for hypertension, prediabetes, hyperlipidemia, vitamin D deficiency, and depression. His clinical course was notable for a five-year history of chronic unsheltered homelessness associated with prolonged standing, walking long distances, and the inability to elevate his lower extremities. Adherence to wound care, medications, and medical appointments was inconsistent due to transportation barriers, limited access to hygiene resources, and competing priorities associated with unsheltered living, contributing to repeated emergency department visits. 

**Figure 1:**
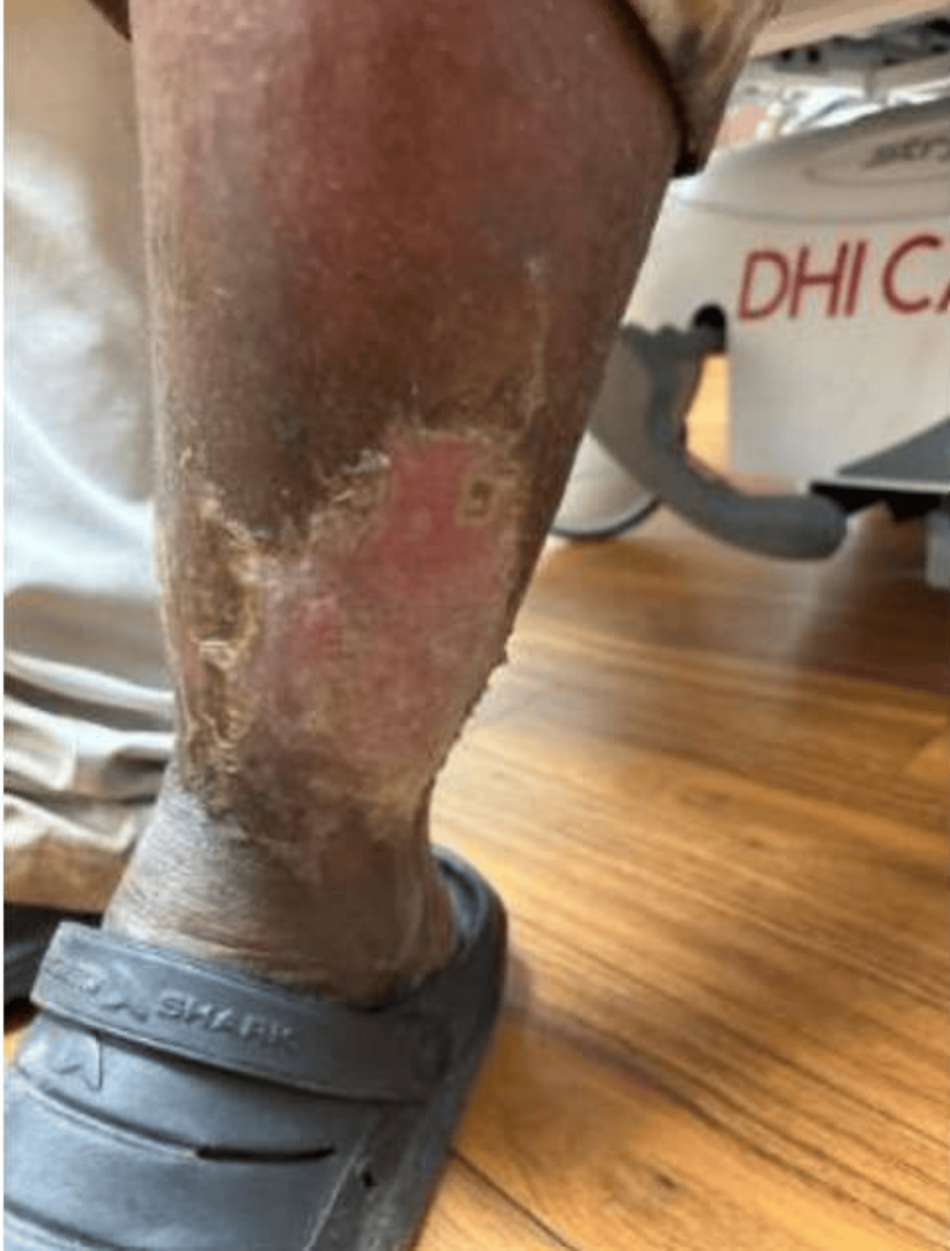
Stasis Dermatitis With Cellulitis During Unsheltered Homelessness (July 31) Clinical image obtained while the patient was living unsheltered, demonstrating erosive changes, inflammation, and skin breakdown consistent with stasis dermatitis, with superimposed cellulitis, concerning for progression to diffuse ulceration.

In late July through early August, the patient was admitted to the hospital for five days due to chronic venous insufficiency complications. At this point in time, the patient was living unsheltered. Bilateral lower legs demonstrated chronic venous stasis changes, including lymphedema, hyperpigmentation, scaly plaques, and lipodermatosclerosis, with evidence of prior ulcerations and scarring. The left lower leg demonstrated erythema, warmth, and tenderness with overlying scale, crust, erosive changes, and purulent drainage, concerning for cellulitis (Figure 1). Risk for progressive ulceration was noted. Venous Doppler ultrasound was negative.

Laboratory studies demonstrated elevated inflammatory markers without leukocytosis (Table 1).

**Table 1:** Laboratory Findings Laboratory values obtained during the patient’s clinical course. Reference ranges and units are provided for each parameter. The dates provided are within the same calendar year. ESR, erythrocyte sedimentation rate; CRP, C-reactive protein; WBC, white blood cell count.

Lab (Units)	Normal Range	July 31: Hospital Admission	August 2: Hospital Admission	August 26: Emergency Department Visit	August 29: Follow Up Appointment
ESR (mm/hr)	0 – 20	25	-	15	-
WBC (X 10^9^/L)	3.8 – 10.8	10.3	10	9.9	7.8
CRP (mg/dL)	<0.50	1.37	-	1.03	-

While hospitalized, the patient was treated with intravenous antibiotics including ceftriaxone and vancomycin for his superimposed bacterial infection. He was discharged with oral cephalexin and trimethoprim-sulfamethoxazole DS for 10 days. A week later, the patient was prioritized for placement at a low barrier emergency shelter for medically vulnerable seniors, defined as people aged 50 and older. To note, PEH aged 50, and even younger, may experience increased rates of frailty and mortality compared to housed counterparts [6]. This includes early onset cognitive decline and a high burden of functional limitations, including difficulty with activities of daily living, falls, visual impairment, depression, chronic pain, and incontinence, amongst other conditions. 

**Figure 2:**
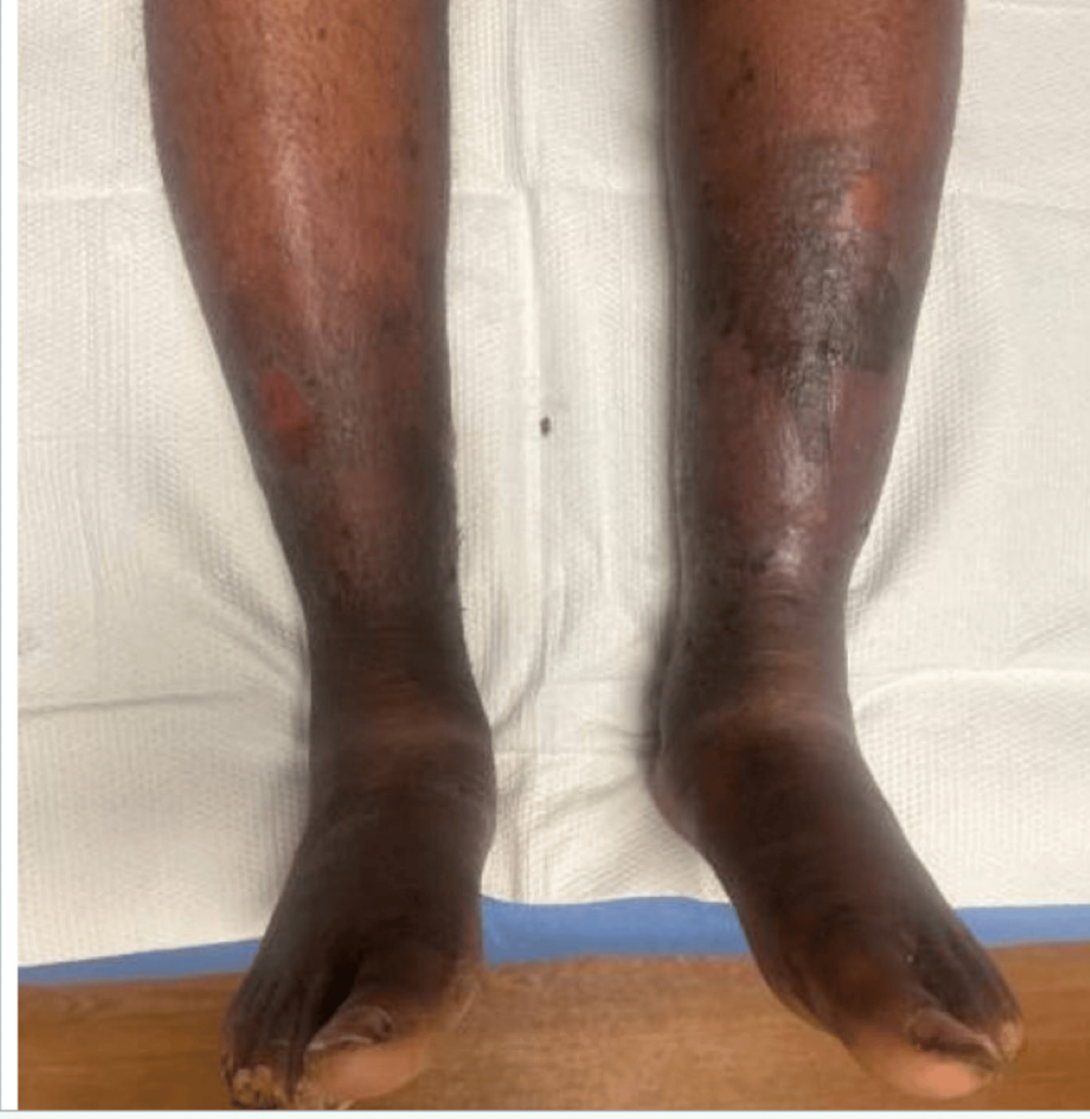
Improvement in Stasis Dermatitis After Placement in the Emergency Shelter (August 26) Clinical image obtained after the patient was housed in an emergency shelter. Bilateral lower legs continued to demonstrate chronic venous stasis changes. No purulence, edema, or ulcerations were noted.

While in the emergency shelter, the patient received dermatologic care consisting of calcium alginate dressings and compression therapy, including Unna boots. At the end of August, while awaiting permanent supportive housing (PSH) placement, the patient visited the emergency department once more due to leg pain secondary to his chronic venous insufficiency. However, he was not admitted, and it was noted that his legs were improving from his prior admission less than a month before. Bilateral lower legs continued to demonstrate chronic venous stasis changes. No purulence, edema, or ulcerations were noted (Figure 2).

At a follow-up appointment after his hospitalization and emergency department visit, his white blood cell count continued to trend downward (Table 1). At the end of September, he transitioned to a PSH unit, an evidence-based intervention that combines affordable housing with voluntary support services for chronically homeless individuals, where he continues to receive intensive case management support [7]. 

One month after obtaining PSH, the patient demonstrated significant dermatologic improvement. Compared to his prior disease course, his chronic venous insufficiency with stasis dermatitis and ulceration showed reduced edema, decreased exudate, and clear progression toward re-epithelialization. Stable housing and case management support enabled reliable access to hygiene facilities, adequate nutrition, safe medication storage, transportation to appointments, and behavioral health support, resulting in markedly improved adherence to dermatologic care. To date, the patient has had no additional emergency department visits, has established care with a primary care physician, and remains in stable condition.

## Discussion

This case illustrates how housing instability in a highly cost-burdened housing market has the potential to undermine chronic disease management, even when appropriate medical therapies are prescribed. In cities such as Orlando, Florida, significant inequities in health care access, driven by lack of insurance, housing insecurity, and food insecurity, are evident along racial and socioeconomic lines [8].

In the Orlando metropolitan area, point-in-time counts indicate that thousands of individuals experience homelessness on any given night. In 2025, approximately 2,781 people were identified as homeless across the region, with 1,090 living unsheltered on the night of the count [9,10]. Unsheltered homelessness in this area has increased significantly compared to previous years, indicating that many individuals remain vulnerable to street-living conditions that complicate chronic disease management, particularly among older adults, with 24% of individuals being 55 years of age or older [11]. Additionally, local service networks, including more than 65 partner agencies through the Homeless Services Network of Central Florida, provide outreach, shelter, and case management services [10]. Between mid-2023 and mid-2024, nearly 14,600 individuals sought assistance from these networks, reflecting the scale of need. These services also support health insurance enrollment and assistance with obtaining essential documentation, such as government-issued identification.

Despite these efforts, substantial barriers to healthcare access persist. Many PEH lack health insurance in this Medicaid non-expansion state, which limits access to care and contributes to adverse health outcomes [12]. These disparities are compounded by structural barriers to specialty care. PEH often lack government-issued identification, which is required to verify in-county residency and qualify for the free specialist referral network (in Orange County, Florida) [13]. Consequently, uninsured patients without identification are frequently unable to access specialty care. For insured patients, follow-up is arranged through their insurance plans; however, co-payments may still prevent attendance at visits. Dermatologic conditions are particularly vulnerable to the effects of homelessness because they often require sustained daily care, environmental control, and longitudinal follow-up [1,2,14].

From an SDOH perspective, the patient encountered multiple structural barriers that directly impeded effective management of chronic venous insufficiency with stasis dermatitis and ulceration [8]. Care was delivered within a fragmented healthcare system, characterized by repeated emergency department visits, intermittent inpatient hospitalizations, and sporadic outpatient encounters at a free clinic, rather than sustained longitudinal care, including specialty care. Transportation insecurity limited attendance at follow-up appointments, and unsheltered living conditions restricted access to hygiene facilities necessary for regular wound cleansing and dressing changes. The absence of safe storage for medications, compression garments, and wound supplies further contributed to inconsistent use of compression therapy, which is essential for venous insufficiency management [1]. Leg elevation, another key component of venous insufficiency management, was effectively unattainable due to the patient’s inability to sleep in a supine position or elevate his lower extremities for sustained periods. This limitation was compounded by the criminalization of unsheltered sleeping in Orlando, Florida (FL Statute 125.0231), as well as hostile architectural designs in public spaces that restrict the use of benches or other surfaces for sleeping [1,15]. Food insecurity and limited access to adequate nutrition likely contributed to delayed wound healing [16]. Separately, chronic psychosocial stress and unmet behavioral health needs may have promoted inflammation and undermined adherence to treatment [17]. Collectively, these SDOH created circumstances in which evidence-based dermatologic interventions could not be reliably implemented, resulting in recurrent infection, delayed healing, and continued reliance on episodic care.

In our case, meaningful and sustained improvement was observed after PSH placement. Although chronic venous ulcers typically heal within 6-12 months with appropriate compression therapy and wound care, depending on ulcer and patient factors, our patient had experienced years of nonhealing ulcers despite intermittent prior treatment [18]. The dramatic clinical shift, characterized by reduced edema, decreased exudate, and progression toward re-epithelialization, suggests that housing stabilization enabled better medical adherence and healing. This case adds to the literature by suggesting that in advanced chronic disease, social interventions such as housing may become an essential component of treatment rather than adjunctive care.

PSH can play an integral role in addressing SDOH and barriers to health care, healing, and improvement. Evidence demonstrates that PSH reduces homelessness, increases housing tenure, and decreases ED visits and hospitalizations [19]. This applies to patients with both moderate- and high-support needs [20]. Although PSH compare to usual social services for effects on psychiatric symptoms, substance use, income, and employment outcomes, the quality of life and community integration scores are significantly higher for participants in PSH [20,21]. Furthermore, data have shown that PSH and Housing First initiatives, with appropriate social resources, actually are cost-effective, with economic benefits from reduced ED visits, hospitalizations, and shelter use [20,21]. 

## Conclusions

Housing status plays an important role in health outcomes. In this case, stable housing and case management support improved adherence to dermatologic treatment, leading to marked improvement in venous stasis dermatitis, as evidenced by reduced edema, decreased exudation, and increased re-epithelialization.

This case highlights the critical role of housing in healthcare. Dermatologists and other specialty providers should consider housing status when managing chronic, non-healing conditions and view referral to housing stabilization programs as an adjunct to medical therapy. Collaboration with allied health professionals may further improve outcomes. Integrating SDOH-focused interventions into care may improve healing, reduce recurrence, and decrease reliance on emergency departments and episodic care.

## References

[1] Yosipovitch G, Nedorost ST, Silverberg JI, Friedman AJ, Canosa JM, Cha A (2023). Stasis dermatitis: an overview of its clinical presentation, pathogenesis, and management. Am J Clin Dermatol.

[2] Gallagher K, Talasila S, Bistline A, Krain R, Ramani L, Jones E (2024). Addressing dermatologic concerns and teledermatology in undomiciled and sheltered populations. Arch Dermatol Res.

[3] Lanham JS, White P, Gaffney B (2022). Care of people experiencing homelessness. Am Fam Physician.

[4] Nilsson SF, Ali Z, Laursen TM (2023). Association of homelessness and skin conditions: a Danish population-based cohort study. Br J Dermatol.

[5] Garcia C, Doran K, Kushel M (2024). Homelessness and health: factors, evidence, innovations that work, and policy recommendations. Health Aff (Millwood).

[6] Mantell R, Hwang YI, Radford K, Perkovic S, Cullen P, Withall A (2023). Accelerated aging in people experiencing homelessness: a rapid review of frailty prevalence and determinants. Front Public Health.

[7] (2026). Permanent supportive housing. https://endhomelessness.org/resources/toolkits-and-training-materials/permanent-supportive-housing/.

[8] Vrtikapa K, Hoque Urmy F, Hoque F (2025). Social determinants of health: the impact of this overlooked vital sign. J Brown Hosp Med.

[9] Caraballo LH (2026). Homelessness count: 40% of Orlando Metro’s unhoused people are children, seniors. https://www.cfpublic.org/housing-homelessness/2025-04-04/homeless-count-40-of-orlando-metros-unhoused-people-are-children-seniors?WMFESocialShare=email.

[10] (2026). Homeless Services Network of Central Florida. https://www.hsncfl.org/.

[11] Zizo LM Christie (2026). Homelessness in Metro Orlando holds steady, but is ‘literally killing’ seniors, advocates say. https://www.clickorlando.com/news/local/2025/04/04/homelessness-in-metro-orlando-holds-steady-but-is-literally-killing-seniors-advocates-say/.

[12] Donohue JM, Cole ES, James CV, Jarlenski M, Michener JD, Roberts ET (2022). The US Medicaid program: coverage, financing, reforms, and implications for health equity. JAMA.

[13] (2026). Eligibility. https://www.orangecountyfl.net/FamiliesHealthSocialSvcs/OrangeCountyMedicalClinic/Eligibility.aspx.

[14] Knapp AP, Rehmus W, Chang AY (2020). Skin diseases in displaced populations: a review of contributing factors, challenges, and approaches to care. Int J Dermatol.

[15] (2026). Statutes & constitution. https://www.leg.state.fl.us/Statutes/index.cfm?App_mode=Display_Statute&Search_String=&URL=0100-0199/0125/Sections/0125.0231.html..

[16] Grada A, Phillips TJ (2022). Nutrition and cutaneous wound healing. Clin Dermatol.

[17] Sun MD, Rieder EA (2021). Psychosocial stress and mechanisms of skin health: a comprehensive update. J Drugs Dermatol.

[18] Raffetto JD, Ligi D, Maniscalco R, Khalil RA, Mannello F (2020). Why venous leg ulcers have difficulty healing: overview on pathophysiology, clinical consequences, and treatment. J Clin Med.

[19] Rog DJ, Marshall T, Dougherty RH, George P, Daniels AS, Ghose SS, Delphin-Rittmon ME (2014). Permanent supportive housing: assessing the evidence. Psychiatr Serv.

[20] Aubry T, Bloch G, Brcic V (2020). Effectiveness of permanent supportive housing and income assistance interventions for homeless individuals in high-income countries: a systematic review. Lancet Public Health.

[21] Jacob V, Chattopadhyay SK, Attipoe-Dorcoo S (2022). Permanent supportive housing with housing first: findings from a community guide systematic economic review. Am J Prev Med.

